# Integrated landscape of copy number variation and RNA expression associated with nodal metastasis in invasive ductal breast carcinoma

**DOI:** 10.18632/oncotarget.26386

**Published:** 2018-12-07

**Authors:** Michael Behring, Sadeep Shrestha, Upender Manne, Xiangqin Cui, Agustin Gonzalez-Reymundez, Alexander Grueneberg, Ana I. Vazquez

**Affiliations:** ^1^ Department of Epidemiology, University of Alabama at Birmingham, Birmingham, AL 35294, USA; ^2^ Comprehensive Cancer Center, University of Alabama at Birmingham, Birmingham, AL 35294, USA; ^3^ Department of Pathology and Surgery, University of Alabama at Birmingham, Birmingham, AL 35294, USA; ^4^ Biostatistics Department, University of Alabama at Birmingham, Birmingham, AL 35294, USA; ^5^ Department of Epidemiology and Biostatistics, Michigan State University, East Lansing, MI 48824, USA; ^6^ Institute for Quantitative Health Science and Engineering, Michigan State University, East Lansing, MI 48824, USA

**Keywords:** breast cancer, lymph node metastasis, integrated genomics, copy number variation, differential expression

## Abstract

**Background:**

Lymph node metastasis (NM) in breast cancer is a clinical predictor of patient outcomes, but how its genetic underpinnings contribute to aggressive phenotypes is unclear. Our objective was to create the first landscape analysis of CNV-associated NM in ductal breast cancer. To assess the role of copy number variations (CNVs) in NM, we compared CNVs and/or associated mRNA expression in primary tumors of patients with NM to those without metastasis.

**Results:**

We found CNV loss in chromosomes 1, 3, 9, 18, and 19 and gains in chromosomes 5, 8, 12, 14, 16-17, and 20 that were associated with NM and replicated in both databases. In primary tumors, per-gene CNVs associated with NM were ten times more frequent than mRNA expression; however, there were few CNV-driven changes in mRNA expression that differed by nodal status. Overlapping regions of CNV changes and mRNA expression were evident for the *CTAGE5* gene. In 8q12, 11q13-14, 20q1, and 17q14-24 regions, there were gene-specific gains in CNV-driven mRNA expression associated with NM.

**Methods:**

Data on CNV and mRNA expression from the TCGA and the METABRIC consortium of breast ductal carcinoma were utilized to identify CNV-based features associated with NM. Within each dataset, associations were compared across omic platforms to identify CNV-driven variations in gene expression. Only replications across both datasets were considered as determinants of NM.

**Conclusions:**

Gains in *CTAGE5*, *NDUFC2*, *EIF4EBP1*, and *PSCA* genes and their expression may aid in early diagnosis of metastatic breast carcinoma and have potential as therapeutic targets.

## INTRODUCTION

In most metastatic carcinomas, the lymph nodes are the first distant organs to be affected. [1]. Approximately half of the 246,000 annual U.S. cases of breast cancer involve women with nodal metastasis (NM) upon diagnosis. Of those without NM at diagnosis, another half will develop distant recurrence and/or relapse [2]. The presence of NM is also a concern in therapeutic and surgical decision making [3]. Yet, in the understanding of metastatic behavior, many of the molecular and genomic changes that occur during the transmission of primary tumor cells to distant sites of propagation remain undiscovered. Metastasis-focused research generally pairs primary-to-distant tumor samples or conflates metastasis, relapse, and death as one outcome in time-to-event studies. We propose that using NM as an alternative endpoint for tumor propagation offers both a point of observation in the metastatic process and a novel method for discovering the genetic underpinnings of NM.

The genomic characteristics of a primary tumor hold structural and functional clues to the behavior of tumor-originating cells in distant organs. There is extensive literature of use of gene expression for the profiling of primary breast tumors in the prediction of metastasis and outcomes [4–12]. Paired primary-to-distant research suggests that the capacity for metastasis is established early in primary tumor growth [13–16] and that distant metastases (nodal and otherwise) show molecular similarities to their primary tumors in both copy number variation (CNV) and mRNA transcription [15, 17–20]. The primary tumors of relapsed NM-negative patients have a higher total CNV burden as compared to relapse-free, NM-negative patients [21]. In NM-free patients, regions of CNV gains associated with a poor prognosis are chromosomes 8 (8p11-12), 11 (11q13-14), and 20 (20q13 33). In NM-positive primary tumors, survival-relevant regions of CNV loss are at 4p, 8p, 9p, 11q, 16q, 17p, and 18p, and areas of gains are at 1q, 8q, 16p, 17q, 19p, and 20q [21–27]. Yet, the findings are limited either by small sample size or by unknown reproducibility in other populations. Our study identifies features in both CNV and transcription platforms, validates findings in a second large dataset, and examines how the two measures interact in ways that are meaningful to NM.

This study presents a validated, gene-level landscape of genomic and intergenic regions associated to NM. We characterized the genomic regions at two levels, cancer-specific CNVs and mRNA expression. Later, we characterized CNV driven changes in mRNA unique to patients with NM. The molecular and genomic features of NM-positive samples were compared to control tumor samples which were MN-negative. To reduce false positives we described this landscape using concordant CNV to mRNA and validated analysis in two large breast cancer cohorts considering potential confounders.

## RESULTS

### Clinical associations to NM

Clinical covariates associations to NM showed a significant relationship between tumor size and NM. In METABRIC, half of the patients without NM had a tumor size ≤20 mm. In TCGA samples, the TNBC molecular subtype was also associated with a decreased instance of NM. Similarly, in the TCGA data, a higher proportion of women with NM were pre-menopausal and slightly younger than their non-NM counterparts. Table 1 shows the association of all covariates with NM in both METABRIC and TCGA.

**Table 1:** Association of nodal metastasis and patient features by data source

	METABRIC			TCGA		
Nodal metastasis present	No	Yes	*p*-value	No	Yes	*p*-value
	(*N* = 389)	(*N* = 383)		(*N* = 293)	(*N* = 357)	
Race			0.021			0.678
- Asian	1 (0.3%)	2 (0.5%)		20 (6.8%)	26 (7.3%)	
- Black	0 (0.0%)	0 (0.0%)		40 (13.7%)	45 (12.6%)	
- missing	225 (57.8%)	179 (46.7%)		22 (7.5%)	36 (10.1%)	
- Other	3 (0.8%)	4 (1.0%)		0 (0.0%)	1 (0.3%)	
- White	160 (41.1%)	198 (51.7%)		211 (72.0%)	249 (69.7%)	
Age at diagnosis (± SD)	59.1 ± 12.2	60.8 ± 14.4	0.088	58.0 ± 12.4	56.1 ± 13.1	
Receptor subtype			0.572			0.043
- missing	0 (0.0%)	0 (0.0%)		29 (9.9%)	23 (6.4%)	
- HER2	28 (7.2%)	34 (8.9%)		9 (3.1%)	21 (5.9%)	
- Luminal	285 (73.3%)	282 (73.6%)		212 (72.4%)	276 (77.3%)	
- TNBC	76 (19.5%)	67 (17.5%)		43 (14.7%)	37 (10.4%)	
ER status			0.708			0.102
- missing	0 (0.0%)	0 (0.0%)		17 (5.8%)	16 (4.5%)	
- negative	104 (26.7%)	108 (28.2%)		88 (30.0%)	84 (23.5%)	
- positive	285 (73.3%)	275 (71.8%)		188 (64.2%)	257 (72.0%)	
PR status			0.171			0.324
- missing	0 (0.0%)	0 (0.0%)		19 (6.5%)	17 (4.8%)	
- negative	184 (47.3%)	200 (52.2%)		110 (37.5%)	121 (33.9%)	
- positive	205 (52.7%)	183 (47.8%)		164 (56.0%)	219 (61.3%)	
HER2 status			0.138			0.591
- missing	0 (0.0%)	0 (0.0%)		96 (32.8%)	114 (31.9%)	
- negative	336 (86.4%)	316 (82.5%)		154 (52.6%)	180 (50.4%)	
- positive	53 (13.6%)	67 (17.5%)		43 (14.7%)	63 (17.6%)	
Menopause status			0.579			0.027
- missing	0 (0.0%)	1 (0.3%)		18 (6.1%)	39 (10.9%)	
- post	293 (75.3%)	291 (76.0%)		213 (72.7%)	228 (63.9%)	
- pre	96 (24.7%)	91 (23.8%)		62 (21.2%)	90 (25.2%)	
Tumor size			<0.0001			<0.0001
- T1 (<20 mm)	230 (59.1%)	126 (32.9%)		115 (39.3%)	72 (20.2%)	
- T2 (>20 <50mm)	157 (40.4%)	234 (61.1%)		160 (54.7%)	229 (64.1%)	
- T3&4 (>50 mm)	2 (0.5%)	23 (6.0%)		17 (5.9%)	56 (15.7%)	
AJCC Stage			<0.0001			<0.0001
- I	251 (64.5%)	3 (0.8%)		115 (39.2%)	8 (2.2%)	
- II	132 (33.9%)	311 (81.2%)		173 (59.0%)	203 (56.9%)	
- III&IV	6 (1.5%)	69 (18.0%)		3 (1.0%)	129 (36.1%)	
- missing	0 (0.0%)	0 (0.0%)		2 (0.7%)	17 (4.8%)	

Abbreviations: HER2, human epidermal growth factor receptor 2; TNBC, triple negative/basal breast subtype; ER, estrogen receptor; PR, progesterone receptor.

^*^Tumor size is AJCC TNM staging.

### Replicated CNV regions of interest (step 1: genomic landscape for NM)

**Figure 1:**
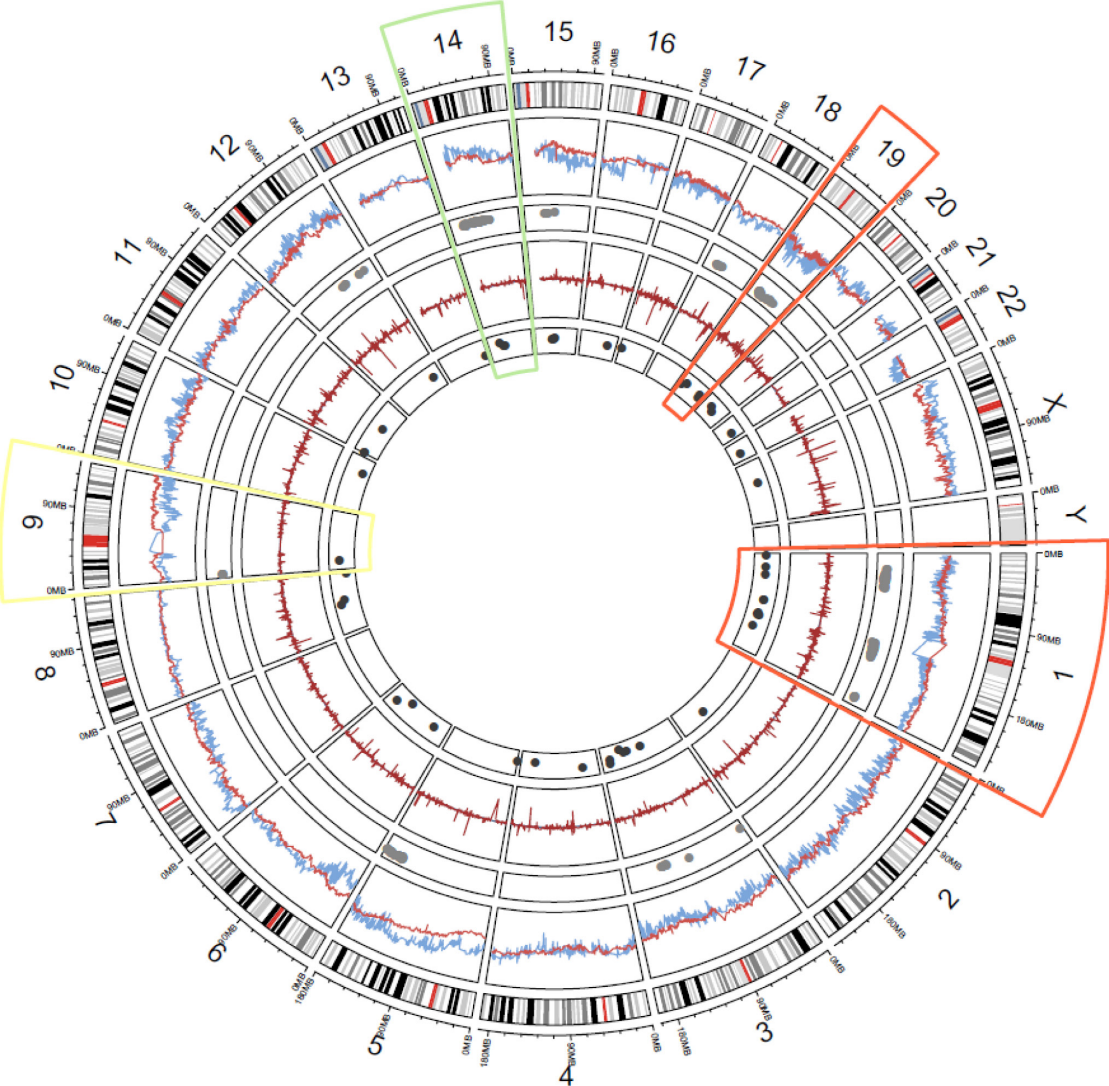
Genome-wide landscape of NM-associated CNV and mRNA: Layers, starting from outermost are (1) chromosome number and mega base scale; (2) ideogram of each chromosome (centromeres in red); (3) genome-wide model coefficient estimates of odds of NM for each gene (METABRIC = blue, TCGA = red); (4) replicated CNV genes associated with NM (grey dots); (5) genome-wide log-fold change between case and control mRNA (METABRIC = blue, TCGA = red); (6) replicated mRNA genes associated with NM (dark grey dots) Highlighted chromosomes bring attention to omic regions of interest (ROIs): orange = consistent CNV/RNA losses in ROIs (chromosomes 1 & 19), white = inconsistent CNV/mRNA measures in replicated ROIs (chromosome 9), green = consistent CNV/mRNA gains in replicated ROIs (chromosome 14).

Among all participants, 450 CNV genes of interest were associated with NM (as determined with a nominal *p*-value α ≤ 0.05) and replicated in both METABRIC and TCGA CNV losses in 314 genes had an association with NM. Specific regions of interest (regions having a density of significantly associated CNVs) were found in large areas of chromosomes 1 (1p32-1p36, 1q21-1q24, and 1q42), 3 (3q11, 3q22-3q26), 9 (9p24), 18 (18q11-18q12), and 19 (19p13 and 19q12). Genes in 1p34 with lowest odds of NM were *AKIRIN1* (OR^METABRIC^ = 0.27, 95% CI 0.10–0.70, OR^TCGA^ = 0.27, 95% CI 0.1–0.59), *NDUFS5* (OR^METABRIC^ = 0.18, 95% CI 0.06–0.53, OR^TCGA^ = 0.28, 95% CI 0.13–0.62), and *RRAGC* (OR^METABRIC^ = 0.18, 95% CI 0.05–0.68, OR^TCGA^ = 0.29, 95% CI 0.13–0.62), (odds ratios are presented in Supplementary Table 1). In 136 genes, per-gene copy gains in CNV measures were associated with NM. There were regions of interest in chromosomes 5 (5q33-5q35), 12 (12q21 & 12q23), 14 (14q11-14q13, 14q21-14q23), and 15 (15q13-15q14, 15q21). Genes with the highest odds of NM were located on chromosome 5 (5q33.1d; *ZNF300*, *CCDC69*, *SLC36A1*, and *SLC36A2*) and 14 (14q 11: in gene *SLC7A8*; 14q13: gene *AKAP6*; and 14q2: gene *CLEC14A*). See Figure 1 and Supplementary Tables 1 and 2. After FDR adjustment for multiple testing, no single gene-level CNV had a significant and replicated association with NM.

### Replicated significant mRNA regions of interest (step 1)

In both TCGA and METABRIC data, 48 genes overlapped with a concordant direction of association to NM. Regions of interest with replicated mRNA losses were found in 23 genes located at chromosomes 1 (1p32-1p36, 1q21, and 1q25), 3 (3q11, 3q22-3q24), 4 (4p32), 5 (5p15), 6 (6q22-6q23), 8 (8q24), 10 (10q23), 11 (11q13), 13 (13q33), 16 (16p11), 19 (19p13 and 19q13), 20 (20q11), and 22 (22q12). The largest decreases in mRNA log-fold change values in both data sets were found in CP (3q24), *HS3ST5* (6q22), *BAI1* (8q24), and *CYP2C8* (10q23). Statistically significant gene-effect changes associated with cases of NM were found for 25 genes across datasets. Regions of interest were in chromosomes 1 (1p13, 1q23), 2 (2q31), 3 (3q26), 4 (4p14), 6 (6q24), 8 (8q21), 9 (9p21), 11 (11p15), 12 (12q24), 14 (14q12-14q13, 14q21), 15 (15q15, 15q21), 16 (16q22), 17 (17p13), 19 (19p13, 19q13), 20 (20p11), 21 (21q22), and X (Xp11). RNA transcripts with the highest log-fold change across datasets were DLX1 (2q31), TMEM156 (4p14), NOVA1 (14q12), and SHC4 (15q21). The lowest nominal *p*-value for log fold change was for *NOVA1*, 0.015 in METABRIC and 0.002 in TCGA. See Supplementary Tables 3 and 4 for details. While coding regions were not significant after FDR correction and validated across data sets, non-coding analysis in TCGA data found three regions significant at FDR 0.1; 6q24.1-6q23.3, 11q13.1, and 15q15.3 - 15q21.1.

### Confirmed links between copy number and transcription (step 2: CNV to mRNA analysis)

**Figure 2:**
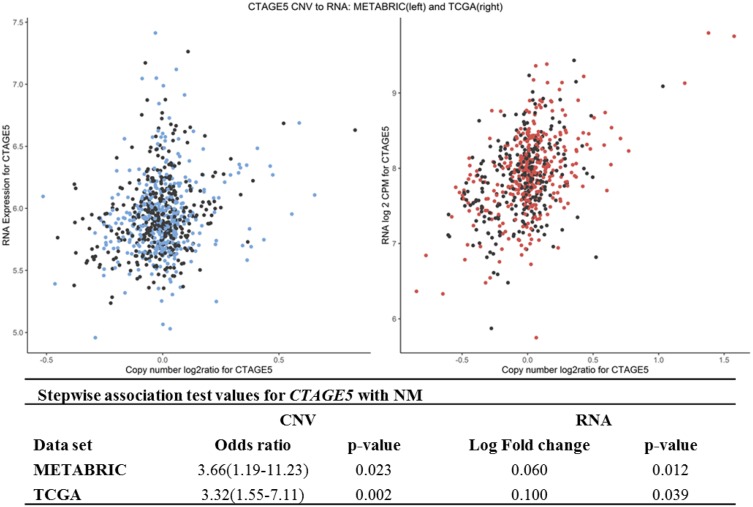
CNV to mRNA relationship in CTAGE5 across both datasets, including adjusted odds of NM and log-fold change between NM-positive and NM-negative samples Black points are NM-negative; red and blue points are NM-positive.

A validated association between CNV and RNA was discovered in *CTAGE5*. For this gene, CNV copy gains and increased RNA were associated with NM-positive patients (Figure 2). CNV-driven loss of expression was found in the *CRELD1* gene (Supplementary Table 5, Supplementary Figure 4). CNV-driven mRNA gains were present in chromosomes 8, 11, 17, and 20. CNV-based upregulation of several genes in chromosome 8 in region 8q23-24: *PSCA*, *SLC30A8*, and *ZFPM2* were evident in approximately 6% of all METABRIC NM-positive patients and in 41% of all TCGA cases (Supplementary Table 6 and Supplementary Figure 5). In cases of NM, multiple genes in the chromosome 17q12-q21 region (*CDC6*, *PSMD3*, and *STARD3* among them) showed a significant relationship of concordant CNV to RNA changes (Supplementary Figures 6–8). In both METABRIC and TCGA data, 20–25% of all women with NM had CNV copy gains or losses that were associated with same-direction RNA changes for this region. A similar result was found for *EIF4EBP1* (8p12) and *NDUFC2* (11q14) (Supplementary Table 7 and Supplementary Figures 9, 10).

### Additional validation measures

Using only TCGA data and the results list from CNV-to-mRNA association in step 2, we performed a preliminary validation of CNV-driven results with two additional omics of protein and methylation. In general, genes with increased CNV-to-mRNA had an inverse relationship with CNV-to-methylation levels (HM450), and a positive correlation with CNV-to-protein levels. Additional omic information was available for four genes; *CRELD1*, *EIF4EBP1*, *PSMD3*, and *STARD3*. Only *CRELD1* and *EIF4EBP1* had significant changes in both protein and methylation which were unique to NM status. For both *CRELD1* and *EIF4EBP1*, NM positive women had CNV-correlated protein increases (Pearson *p*-value of < 0.001 for both). In the same genes, methylation was inversely correlated to CNV (Pearson *p*-value of 0.02 and 0.02 respectively) (See Supplementary Table 8). In the top results from validated CNV-driven mRNA changes, we found the choline metabolism pathway in KEGG cell signaling (hsa05231), to be enriched in NM positive patients.

## DISCUSSION

This investigation utilized TCGA and METABRIC data to identify and replicate genomic and transcriptional features in association with NM in ductal breast tumors. While we proposed NM as a proxy for metastatic behavior in general, the main objective of this paper is the creation of a descriptive landscape of omics in primary tumors with metastatic behavior. NM however is limited when considered as the outcome. While the overall objective of treatment is to achieve metastasis-free survival, rather than preventing nodal metastasis, NM has several advantages for the analysis, e.g., NM is cross-sectional and does not need follow up. Identified genes or other non-coding genomic regions will contribute to the understanding of the metastatic process. The genome-wide CNV association study revealed more than 400 gene-level areas of amplification and deletion that were replicated in both sets of data. Similar mRNA association testing gave a set of 48 gene-level transcripts consistently associated with NM status. Both sets of results were obtained without correction for multiple testing in order to be lax and reduce the number of false negatives. Genes were prioritized for discussion using the top results from statistical analysis in step 1(integrated CNV and mRNA landscape); significant genes (nominal *p*-value α ≤ 0.05) were then tested in step 2, CNV-to-mRNA genome-wide association testing. From these results, per-gene gains in CNV measurement had translational effects in the 8q12, 11q13-14, 14q, 20q1, and 17q14-24 regions. There were transcriptional corollaries across areas of CNV copy loss in chromosomes 1p and 1q and in 19p13. In chromosome 3 (3p25), there was a slight CNV-driven loss of transcription linked to *CRELD1*.

Chromosome 14 was of interest in regard to replicated CNV gains in the *CTAGE5* gene (14q21). This gene is a member of the CTAGE family, and a variety of tumors, including those of the breast, express *CTAGE5* exclusively [28]. The protein for this gene is involved in collagen VII transport in the endoplasmic reticulum [29]. The relationship between collagen density and tumorigenesis in mouse primary tumors and lymph nodes has been examined [30–32]. Although most research has focused on collagen I, collagen VII has been associated with *in situ* tumors in some cases of breast cancer [33]. An additional quality of *CTAGE 5* is tumor-specific splicing [34], with an example of gene fusion in prostate cancer [35]. *CTAGE5*, as an antigen specific to tumors, has promise as a therapeutic target.

The gene for prostate stem cell antigen (*PSCA*) on chromosome 8 (8q24) had significantly associated CNV-to-mRNA expression only in cases of NM. For various tumor types, there was increased expression of this gene. For pancreatic and bladder cancers, both expression and copy number increase with metastases [36, 37]. For Asian populations, mutations in *PSCA* are linked to an increase in breast cancer risk, with an increased risk of NM. [38, 39] The genes *NDUFC2* and *EIF4EBP1* in 11q14 and 8q12 are amplified driver genes in several cancers [40–42], yet their link to NM in breast cancer is novel (Supplementary Figures 9, 10).

CNV-based genome-wide analysis of non-coding regions revealed three significant results after FDR correction. It is difficult to differentiate between protein coding and non-coding effects in these regions. In many patients the CNVs are large and involve both coding and non-coding regions. Loss of heterozygosity (LOH) at *RAD51* 15q14-15 loci and 6q23-24 at *SASH1* loci in breast cancer has been linked to poor outcomes [43–45]. Furthermore, previous breast cancer research suggests that the consequences of CNV change on noncoding RNA seems to be less frequent than in protein coding and non-coding regions [46]. CNV-driven mRNA genes associated with NM were found to be enriched in the choline kinase pathway. Choline kinase has been observed as overexpressed in approximately 40% of breast tumors [47], and has been evaluated as a promising imaging tracer for breast tumors diagnosis [48, 49]. In Prostate cancer, choline PET/CT has been used successfully to detect recurrence and lymph node staging [50], and recent research in breast cancer has suggested a similar choline-based diagnosis strategy as promising [51, 52]. However, the relationship between choline and NM, as well as its association with CNV, presents novel topics of further research.

Relative to copy number aberrations and their mRNA consequences, we found more validation of per-gene CNV regions associated with NM than for the per-gene mRNA approach or CNV-driven mRNA analysis. This can be expected from a per-gene CNV approach, since mapping multi-gene segment units of copy number to an individual gene gives a distortion in interpreting measures of frequency. Empirically, approximates of per-gene transcription as correlated to CNV in all cancers suggest that ~60% [53] of the associated mRNA should be based in CNV. Breast cancer-specific studies of the CNV effect upon expression suggest ~12% concordance [54]. Our results show ~10% of CNV changes altered mRNA in a meaningful way for NM. Rather than predicting tumor versus normal, we examined NM within the situation of cancer. Therefore, expected proportions may not apply. It is important to note that the two steps of the analysis will not necessarily yield overlapping results, as they are geared towards different measures; The first step constructs a landscape of gene-level odds of NM for both CNV and mRNA measures while the second step identifies CNV-driven changes in mRNA unique to patients with NM. The strength of the second stage of the analysis (step 2) is built around the ability to validate gene-level, CNV-associated changes in mRNA. Differing approaches to CNV calls between sets of data may limit the effectiveness of our validation approach. A low rate of validation is expected; residual influences on validity, such as measurement errors, selection bias, and target population, would lead to spurious findings unique to both METABRIC and TCGA. Under the null hypothesis (i.e., if the gene has no effect on nodal metastasis), there will be at least 5% of false positives just from chance; however, the same false positive has a chance to be significant again only in the 0.25% of the tests. Finally, the limitation of lymph node metastasis as a proxy for overall metastatic behavior should be considered. Many cancers have alternate routes to distant metastasis, other than lymph nodes.

Our analysis found examples of CNV-driven relationships to mRNA that were unique to single sets of data, but they were not reproduced. In TCGA, the 15q21.1a region of chromosome 15 had the strongest statistical association with outcome. CNV unit increases in the gene *SEMA6D* had high odds of NM combined with a significant mRNA fold change in NM patients. Yet the closest significant regions of interest in METABRIC data were more than 1,000 kpb away from this result, and there was no clear link between CNV and mRNA. Large regions of CNV loss in chromosome 1 were validated across both sets of data, yet had few transcriptional correlates. Traditionally, joint analyses of copy number and expression data are used to guide internal validity through the discovery of CNV-driven mRNA effects [55–58].

## MATERIALS AND METHODS

### Patients and samples

Cancer genome data were obtained from two independent cohorts. The Molecular Taxonomy of Breast Cancer International Consortium (METABRIC) data (*N* = 772) were acquired from Synapse DREAM7 Breast Cancer Prognosis Challenge, already processed according to the METABRIC source paper [56]. A second set of data (*N* = 650) came from The Cancer Genome Atlas (TCGA) Data Portal [59]. TCGA level 3 data are also open-access and pre-processed. Since lobular breast cancer differs from ductal cancer in biological characteristics, indolence, and metastatic behavior, samples were exclusively from invasive ductal carcinomas [60, 61]. All patients were female, with no history of a prior malignancy or of neoadjuvant treatment. The response variable of NM was defined for both datasets using TNM pathologic staging [62] for lymph nodes (N). All TNM *N* values of 0 were considered controls (NM 0); *N* values greater than 0 were considered cases. Patient demographics and characteristics are described in Table 2 and Supplementary Figure 1.

**Table 2:** Patient features by data source

Features	METABRIC	TCGA
	(*N* = 772)	(*N* = 650)
Nodal metastasis present		
- No	389 (50.4%)	293 (45.1%)
- Yes	383 (49.6%)	357 (54.9%)
Race		
- Asian	3 (0.4%)	46 (7.1%)
- Black	0 (0.0%)	85 (13.1%)
- missing	404 (52.3%)	58 (8.9%)
- Other	7 (0.9%)	1 (0.2%)
- White	358 (46.4%)	460 (70.8%)
Age at diagnosis (± SD)	59.9 ± 13.4	56.9 ± 12.8
Receptor subtype		
- missing	0 (0.0%)	52 (8.0%)
- HER2	62 (8.0%)	30 (4.6%)
- Luminal	567 (73.4%)	488 (75.1%)
- TNBC	143 (18.5%)	80 (12.3%)
ER status		
- missing	0 (0.0%)	33 (5.1%)
- negative	212 (27.5%)	172 (26.5%)
- positive	560 (72.5%)	445 (68.5%)
PR status		
- missing	0 (0.0%)	36 (5.5%)
- negative	384 (49.7%)	231 (35.5%)
- positive	388 (50.3%)	383 (58.9%)
HER2 status		
- missing	0 (0.0%)	210 (32.3%)
- negative	652 (84.5%)	334 (51.4%)
- positive	120 (15.5%)	106 (16.3%)
Menopause status		
- missing	1 (0.1%)	57 (8.8%)
- post	584 (75.6%)	441 (67.8%)
- pre	187 (24.2%)	152 (23.4%)
Tumor size^*^		
- T1 (<20 mm)	356 (46.1%)	187 (28.9%)
- T2 (>20 <50 mm)	391 (50.6%)	389 (59.9%)
- T3&4 (>50 mm)	25 (3.2%)	73 (11.2%)
AJCC Stage		
- I	254 (32.9%)	123 (18.9%)
- II	443 (57.4%)	376 (57.8%)
- III&IV	75 (9.7%)	132 (20.3%)
- missing	0 (0.0%)	19 (2.9%)

Abbreviations: HER2, human epidermal growth factor receptor 2; TNBC, triple negative/basal breast subtype; ER, estrogen receptor; PR, progesterone receptor.

^*^Tumor size is AJCC TNM staging.

### Copy number and transcriptome data

METABRIC and TCGA used Affymetrix Genome-Wide SNP array 6.0 to derive somatic copy number variations (CNVs). METABRIC preprocessing identified somatic CNV segments in tumors using the HMM-Dosage method [63]. A similar patient-by-gene matrix was created with TCGA data using normalized circular binary segmentation [64] files for each patient. A mean log2 ratio per segment was assigned to each genic and intergenic region within the segment according to METABRIC annotation. METABRIC used the Illumina HT-12v3 platform in gene expression analysis. Pre-processing included spatial artifact correction, summarization, and normalization of log2 intensities with bead-array and BASH R packages [65, 66]. In TCGA, normalized mRNA expression counts were derived from the TCGA Level 3 RNAseqV2 expression data. Illumina HiSeq 2000 was used to create the TCGA transcriptional data.

### Association studies and omic integration

This study was performed in two steps (see workflow in Supplementary Figure 2). In the first step of the analysis, we created genome-wide landscapes of NM associated CNV and mRNA in both TCGA and METABRIC, and then integrated both CNV and mRNA results. This analysis was done for coding regions in TCGA and METABRIC and non-coding regions in TCGA. Non-coding analysis was done only in TCGA, since data was not available in METABRIC. Genome-wide, covariate-adjusted association tests were done at a gene level unit of analysis, separately evaluating the association between the levels first of CNV, and then mRNA, with the response variable yes/no NM. In total, association results were produced for five separate sets (see Supplementary Figure 3): TCGA protein coding CNV, TCGA mRNA, METABRIC protein coding CNV, METABRIC mRNA, and TCGA non-coding CNV. Equations for genome wide tests are:

$$\ln\;\left(\frac{P(y_{i}\;=\;1)}{1-P(y_{i}=\;1)}\right)\;=\;\eta_{ij}$$

Where *y_i_* is a dummy variable representing subject's *i*-th NM status (yes = 1, no = 0), and η_*ij*_ is a linear predictor of the form:

$$\eta_{ij}=\;\mu +X_{i}\beta +O_{ij}\gamma$$

Where *μ* is a common intercept, *X_i_* is the *i*-th row of the incidence matrix *X* representing different sets of covariates for each data set (METABRIC and TCGA), *β* is the vector of corresponding effects; *O*_ij_ is the intensity of the *j*-th feature of the omic *O* in the *i*-th subject, and γ the corresponding effect. For METABRIC data, the columns of *X* consisted of grade, tumor size, age at the moment of diagnosis, and race. For TCGA data, they consisted of molecular subtype, tumor size, age at diagnosis, and race.

In the second step of the analysis we regressed a CNV to mRNA on a gene-by-gene basis [55, 58]. The analysis was conducted to examine the consequence of per-gene CNV gain/loss upon mRNA within the same sample. To identify the modifying effect of NM upon CNV related changes in expression, both datasets were stratified by NM status, and the following tests were performed with the iGC Bioconductor package [67]. Gene expression driven by CNV was identified first by grouping all per-gene CNVs as copy gains (log2 ratio ≥ 0.4), copy losses (log2 ratio ≤ −0.4), and between-threshold values as diploid/neutral (log2ratio null = 0). Thresholds for log2 ratio values were chosen at higher amplitudes than greater than or less than |0.1| of earlier approaches [68, 69] in order to better show gene-level, rather than chromosome-level, or arm-level, CNV events [70]. The variations in gene expression between CNV-gain genes and diploid normals and CNV-loss genes and diploid normals were tested with an unequal variance Student's *t*-test. Filtering of results was based on the false discovery rate (FDR) adjusted *p*-value (α = 0.1) and consistent direction of CNV-to-RNA association. A relaxed *p* value threshold was selected to avoid losing genes that could be false negatives in a stringent testing by the cost of accepting more false positives. CNV-driven gene transcripts unique to NM status were found for both METABRIC and TCGA. Finally, significant genes in both datasets were then identified within each NM group.

Three additional measures of validation were used to supplement our findings. We performed an enrichment analysis on all significant CNV-driven mRNA genes in Enrichr [71]. Using only TCGA data, we checked for CNV-driven changes in the added omic measures of protein and methylation. In order to account for any non-coding CNVs of importance to our outcome, we also examined the association of non-coding regions of CNV to NM status. However this was done only in TCGA, since non-coding data is not available in METABRIC (see Statistical analysis section in Supplementary Materials).

## CONCLUSIONS

In sum, we have identified, in invasive ductal beast carcinomas, CNV-based regions of interest that are associated with NM. Genes in regions 14q21, 8p12, 8q24, 11q14, and various locations on chromosome 1 and 17 may be associated with the development of NM, since the chromosome copy loss/gain happened after the development of NM, and the associated expressions of these genes were different by NM status, suggesting either a role in or a consequence of development of metastases.

## SUPPLEMENTARY MATERIALS FIGURES AND TABLES













## Abbreviations

nM: nodal metastasis
CNV: copy number variation
METABRIC: Molecular Taxonomy of Breast Cancer International Consortium
TCGA: The Cancer Genome Atlas
kbp: kilobase pair
