# Supplementary material for: Integrated landscape of copy number variation and RNA expression associated with nodal metastasis in invasive ductal breast carcinoma
Source: Oncotarget. 2018 Dec 7;9(96):36836–48. doi: 10.18632/oncotarget.26386 (PMC6305147; doi:10.18632/oncotarget.26386)
Supplement: Supplementary file 1 [file oncotarget-09-36836-s001.pdf]

Integrated landscape of copy number variation and RNA expression associated with nodal metastasis in invasive ductal breast carcinoma

SUPPLEMENTARY MATERIALS

Design and participants

In order to examine the effect of CNV on difference in lymph node metastasis, we employed a case-control design. A case was defined as a patient having invasive ductal carcinoma (IDC) and lymph node metastasis at the time of sample collection and diagnosis. All controls were IDC patients with metastasis-free lymph nodes at the time of diagnosis. All statistical analysis was carried out using a set of patients (n = 772) from the Molecular Taxonomy of Breast Cancer International Consortium (METABRIC). The same approach was then carried out in a second large set (n = 650) from The Cancer Genome Atlas (TCGA).

Clinical and omic samples for METABRIC were collected only from breast cancer patients. Data comes from 5 separate sources in the EU and Canada; Cambridge Breast Unit at Addenbrooke's Hospital (Cambridge), Guy's Hospital (London) and Nottingham University City Hospital, the Tumor Bank of British Columbia (Vancouver) and the Manitoba Tumor Bank. METABRIC's sample omics feature copy number variation (Affymetrix SNP 6.0) and expression (Illumina HT 12 array) platforms. METABRIC data was provided by Synapse training dataset in the Breast Cancer Challenge (https://www.synapse.org/#!Synapse:syn1688369/wiki/27311). A total of 772 METABRIC patient samples were included in this analysis. TCGA data is comprised of 650 tumor samples from women with invasive breast carcinoma. Omic information used from TCGA included CNV (Affymetrix SNP 6.0) and mRNA (Illumina HiSeq). Level 3 TCGA RNA Seq files used (rsem.genes.results) measure raw expression signal for a gene. Copy number files used (nocnv.seg) were normalized to remove germline CNV. Since METABRIC array data uses human genome 18 (hg18) as annotation, all downloads were hg18. Institutional Review Board approval was obtained for METABRIC Synapse data. TCGA level 3 data is publicly available.

Inclusion criteria and variable selection: In order to qualify for analysis, samples needed to be exclusively invasive ductal carcinoma. This means that not only were all other histologies (e.g. lobular, colloid, tubular) excluded, but also all non-invasive in-situ tumors were left out. Since METABRIC includes a large portion of stage 0 (*in-situ*), all samples with missing stage were also excluded as a precaution against non-invasive samples. The European Society of Medical Oncology (ESMO) staging [1] uses tumor size, nodal involvement, and presence of distant metastasis (TNM classification [2]) to categorize breast tumors. ESMO stage 0 indicates a non-invasive, localized yet still malignant tissue sample. All patients were female, with no history of prior malignancy or neoadjuvant treatment. Samples with missing information on NM outcome were excluded from analysis. Unless indicated otherwise, variables with over 10% missing values were also excluded from analysis. Since the METABRIC variable of stage was only available for half of the dataset, we chose to exclude all unstaged participants in an effort to avoid possible misclassification of noninvasive tumors.

Clinical variables

The response variable of lymph node metastasis (NM) was defined for both datasets using the previously mentioned TNM pathologic staging for lymph nodes (N). All TNM N values of 0 were considered controls (NM 0). Any N values greater than 0 were considered cases of NM.

Non-omic data from METABRIC includes age at diagnosis, tumor size at largest dimension, grade, stage, histological type, treatment received, menopausal status, lymph nodes positive, total lymph nodes removed cellularity, and Nottingham Prognostic Index (NPI). In addition, receptor status for estrogen, progesterone, and HER2 was assayed using two methods: immunohistochemistry (for 40%–60% of samples) and expression (100% of samples) [3]. Stage and TNM staging variables were recorded according to the AJCC 7th Edition guidelines [4]. Other clinical and pathologic data include: history of previous malignancy, neoadjuvant therapy given, method of diagnosis, surgical procedure, total lymph nodes examined, total lymph nodes positive for metastasis, histologic diagnosis, menopause status, and age at diagnosis. Receptor status was measured by immunohistochemistry for estrogen, progesterone and HER2 receptors. See Table 1 in paper for a full overview of clinical variables shared between both datasets.

Copy number measures

METABRIC and TCGA share the same CNV platform; Affymetrix Genome-Wide SNP array 6.0.

Somatic CNV segments in tumors were identified using The HMM-Dosage method [5]. For CNV alterations on a gene-centric level, annotation files used are Ensembl 54 (hg18) for protein-only probes in Illumina HT-12v3 array. It is important to note that, in METABRIC CNV segment identification; there was not exact matching of tumor to normal pairs. In the discovery set of 997 tumors, 473 normal samples were available for use in a pooled approach. The workflow (not done in this paper) to summarize data in normalized Log2 intensities was accomplished using probe level modelling and normalization with SNP-RMA and aroma-affymetrix software [3]. CNV data for this study comes from Level 3 TCGA Affymetrix Genome-Wide SNP array 6.0. Processing pipeline full details are documented in a Broad Institute GenePattern pipeline [6]. Circular binary segmentation (CBS) [7] was used to create copy number segments, which were then assigned mean log-ratios per segment. This research uses CBS files for each patient to follow the "gene-centric" analysis of CNV used in METABRIC. Annotation files used are Ensembl 54 (hg18) for protein-only probes in Illumina HT-12v3 array.

RNA measures

The Illumina HT-12v3 platform was used in gene expression analysis. Similar to CNV identification, there were 997 tumors matched to 144 normal samples. The resulting workflow included spatial artifact correction, summarization, and normalization of Log2 intensities using beadarray and BASH R packages [8, 9]. In TCGA, Normalized mRNA expression counts are derived from the TCGA Level 3 RNAseqV2 expression data. Illumina HiSeq 2000 was the platform used to create the data, and it was processed by the University of North Carolina to produce counts using Map Splice [10] for alignment and RSEM [11] for quantification.

Statistical analysis

Analysis of association between clinical predictor variables and response (NM) was carried out using a chi square test, or F-test when appropriate. Molecular tests of association were covariate adjusted to account for confounding from variables associated with both exposure and outcome. In METABRIC, final covariates were tumor grade, tumor size, patient age at diagnosis, and race. TCGA covariates included receptor subtype, age at diagnosis, tumor size, and race. These covariates are used in both CNV and RNA association tests for all data.

Genome-wide CNV association tests used logistic regression for each gene in the genome. The logistic model allows for a dichotomous response variable, nodal metastasis (NM, positive or negative) and multiple predictor variables. We corrected for multiple testing in the GWAS analyses using false discovery rate (FDR) methods with a type 1 error set at 0.05. CNV data is given in METABRIC as a gene-by-gene summary of normalized segment means. In TCGA, CNV data is available as a per-patient file of segment means with a start/end location on each chromosome. We summarized the segment intensity data for each gene with the average intensity of each gene using the annotation package GenomicRanges [12]. These files were then merged using the "summarizeOverlaps" function to give a METABRIC-like matrix of patient-by-gene segment means.

RNA data is available in METABRIC as a probe normalized expression value. We converted from probe to gene level using the "CollapseRows" function in the WGCNA package [13]. For each gene in the genome, we fitted a linear model with the R package limma [14]. Empirical Bayes [15] shrinkage was used in calculating a t-statistic for each gene. Multiple comparisons were corrected for using the Benjamini-Hochberg approach. In TCGA, raw counts of RNA per-gene were compiled across the genome of each patient and then assembled per gene matrix using the edgeR R package. [16] Normalization factors for the raw data matrix were calculated, as well as common dispersion values.

Gene lists from CNV and RNA association tests were then reviewed for any significant overlaps, both between datasets and within them. The statistical testing of this involved a contingency table of two gene lists, the whole set of possible genes (entire genome) and Fisher's exact test. The R package GeneOverlap [17] was used to indicate replication of important by-gene CNV or RNA associations. As mentioned in the approach, further evaluation of consistency in direction of effect was also done.

A copy number association analysis [18, 19] was done to examine the effect of per-gene, CNV-related gain/loss upon RNA within the same tumor. To account for differences across NM status, both datasets were split by NM, and the following tests were performed with the iGC Bioconductor package [20]. Gene expression driven by CNV was identified first by grouping all per-gene CNVs as copy gains (log2 ratio ≤ 0.4), copy losses (log2 ratio ≥ -0.4), and between-threshold values as diploid/neutral. The variations in gene expression between CNV-gain genes and diploid normals and CNV-loss genes and diploid normals were measured with an unequal variance Student's t-test. Filtering of results was based on the false discovery rate (FDR) corrected p-value ($\alpha = 0.1$) and consistent direction of CNV-to-RNA association. A relaxed p value threshold was selected to avoid losing genes that could be false negatives in a stringent testing by the cost of accepting more false positives. CNV-driven gene transcripts unique to NM status were found for both METABRIC and TCGA. In a final step of replication,

intersecting genes were then identified across datasets within each NM group.

Two additional validation steps of 1) enrichment analysis and 2) CNV-driven methylation and protein changes in TCGA were performed. Enrichment analysis was performed on all top result CNVs associated with NM, Fischer exact testing was used to indicate the probability of a gene occurring in any set of ontology genes [21]. We utilized the cBIO Portal [22] for additional analysis validating or CNV-driven mRNA results. Using the same TCGA samples in our research, we compared CNV-driven changes in protein and methylation for our CNVs of interest. Omic data was available for four genes; CRELD1, EIF4EBP1, PSMD3, and STARD3. Pearson coefficients were used to measure correlation between CNV and methylation or protein mass-spectrometry for the same sample.

REFERENCES

1. Senkus E, Kyriakides S, Penault-Llorca F, Poortmans P, Thompson A, Zackrisson S, Cardoso F. Primary breast cancer: ESMO clinical practice guidelines for diagnosis, treatment and follow-up. Ann Oncol. 2013; 24. https://doi.org/10.1093/annonc/mdt284.
2. NCI. PDQ® Breast Cancer Treatment. National Cancer Institute. 2013. Available from www.cancer.gov/cancertopics/pdq/treatment/breast/healthprofessional.
3. Curtis C, Shah SP, Chin SF, Turashvili G, Rueda OM, Dunning MJ, Speed D, Lynch AG, Samarajiwa S, Yuan Y, Gräf S, Ha G, Haffari G, et al. The genomic and transcriptomic architecture of 2,000 breast tumours reveals novel subgroups. Nature. 2012; 486:346–52. https://doi.org/10.1038/nature10983.
4. Edge SB, Compton CC. The American Joint Committee on Cancer: the 7th edition of the AJCC cancer staging manual and the future of TNM. Annals of surgical oncology. 2010; 1471–4. https://doi.org/10.1245/s10434-010-0985-4.
5. Ha G, Shah SP. Distinguishing Somatic and Germline Copy Number Events in Cancer Patient DNA Hybridized to Whole-Genome SNP Genotyping Arrays. Methods in Molecular Biology. Springer Science and Business Media, LLC; 2013.
6. Broad Institute. Affymetrix SNP6 Copy Number Inference Pipeline. 2013. Available 2015; 1. from http://www.broadinstitute.org/cancer/software/genepattern/modules/snp6copynumberpipeline.
7. Olshen AB, Venkatraman ES, Lucito R, Wigler M. Circular binary segmentation for the analysis of array-based DNA copy number data. Biostatistics. 2004; 5:557–72. https://doi.org/10.1093/biostatistics/kxh008.
8. Cairns JM, Dunning MJ, Ritchie ME, Russell R, Lynch AG. BASH: A tool for managing BeadArray spatial artefacts. Bioinformatics. 2008; 24: 2921–2. https://doi.org/10.1093/bioinformatics/btn557.
9. Dunning MJ, Smith ML, Ritchie ME, Tavar S. Beadarray: R classes and methods for Illumina bead-based data. Bioinformatics. 2007; 23: 2183–4. https://doi.org/10.1093/bioinformatics/btm311.
10. Wang K, Singh D, Zeng Z, Coleman SJ, Huang Y, Savich GL, He X, Mieczkowski P, Grimm SA, Perou CM, MacLeod JN, Chiang DY, Prins JF, et al. MapSplice: Accurate mapping of RNA-seq reads for splice junction discovery. Nucleic Acids Res. 2010; 38. https://doi.org/10.1093/nar/gkq622.
11. Li B, Dewey CN. RSEM: accurate transcript quantification from RNA-Seq data with or without a reference genome. BMC Bioinformatics. 2011; 12:323. https://doi.org/10.1186/1471-2105-12-323.
12. Lawrence M, Huber W, Pagès H, Aboyoun P, Carlson M, Gentleman R, Morgan MT, Carey VJ. Software for Computing and Annotating Genomic Ranges. PLoS Comput Biol. 2013; 9. https://doi.org/10.1371/journal.pcbi.1003118.
13. Langfelder P, Horvath S. WGCNA: an R package for weighted correlation network analysis. BMC Bioinformatics. 2008; 9:559. https://doi.org/10.1186/1471-2105-9-559.
14. Ritchie ME, Phipson B, Wu D, Hu Y, Law CW, Shi W, Smyth GK. Limma powers differential expression analyses for RNA-sequencing and microarray studies. Nucleic Acids Res. 2015; 43:e47. https://doi.org/10.1093/nar/gkv007.
15. Smyth GK. Linear models and empirical bayes methods for assessing differential expression in microarray experiments. Stat Appl Genet Mol Biol. 2004; 3: Article3. https://doi.org/10.2202/1544-6115.1027.
16. Robinson MD, McCarthy DJ, Smyth GK. edgeR: a Bioconductor package for differential expression analysis of digital gene expression data. Bioinformatics. 2010; 26:139–40. https://doi.org/10.1093/bioinformatics/btp616.
17. Shen LMS. GeneOverlap: Test and visualize gene overlaps. 2013.
18. Parris TZ, Danielsson A, Nemes S, Kovács A, Delle U, Fallenius G, Möllerström E, Karlsson P, Helou K. Clinical implications of gene dosage and gene expression patterns in diploid breast carcinoma. Clin Cancer Res. 2010; 16:3860–74. https://doi.org/10.1158/1078-0432.CCR-10-0889.
19. Pollack JR, Sørlie T, Perou CM, Rees CA, Jeffrey SS, Lonning PE, Tibshirani R, Botstein D, Børresen-Dale AL, Brown PO, Sorlie T, Borresen-Dale AL. Microarray analysis reveals a major direct role of DNA copy number alteration in the transcriptional program of human breast tumors. Proc Natl Acad Sci U S A. 2002; 99:12963–8. https://doi.org/10.1073/pnas.162471999.
20. Lai YP, Wang LB, Wang WA, Lai LC, Tsai MH, Lu TP, Chuang EY. iGC—an integrated analysis package of gene expression and copy number alteration. BMC Bioinformatics. BMC Bioinformatics; 2017; 18:35. https://doi.org/10.1186/s12859-016-1438-2.
21. Chen EY, Tan CM, Kou Y, Duan Q, Wang Z, Meirelles GV, Clark NR, Ma'ayan A. Enrichr: Interactive and collaborative HTML5 gene list enrichment analysis tool. BMC Bioinformatics. 2013; 14. https://doi.org/10.1186/1471-2105-14-128.
22. Cerami E, Gao J, Dogrusoz U, Gross BE, Sumer SO, Aksoy BA, Jacobsen A, Byrne CJ, Heuer ML, Larsson E, Antipin Y, Reva B, Goldberg AP, et al. The cBio cancer genomics portal: an open platform for exploring multidimensional cancer genomics data. Cancer Discov. 2012; 2:401–4. https://doi.org/10.1158/2159-8290.CD-12-0095.

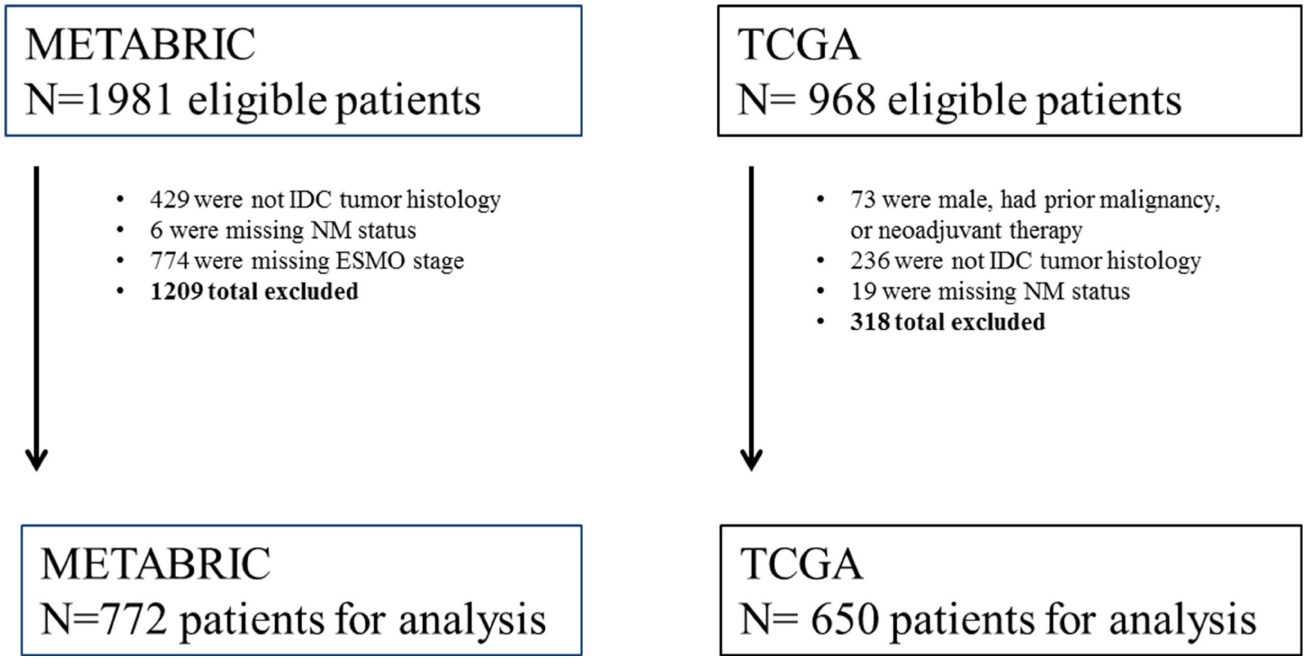

Supplementary Figure 1: CONSORT-like flow diagram showing reason for exclusion in both METABRIC and TCGA datasets.

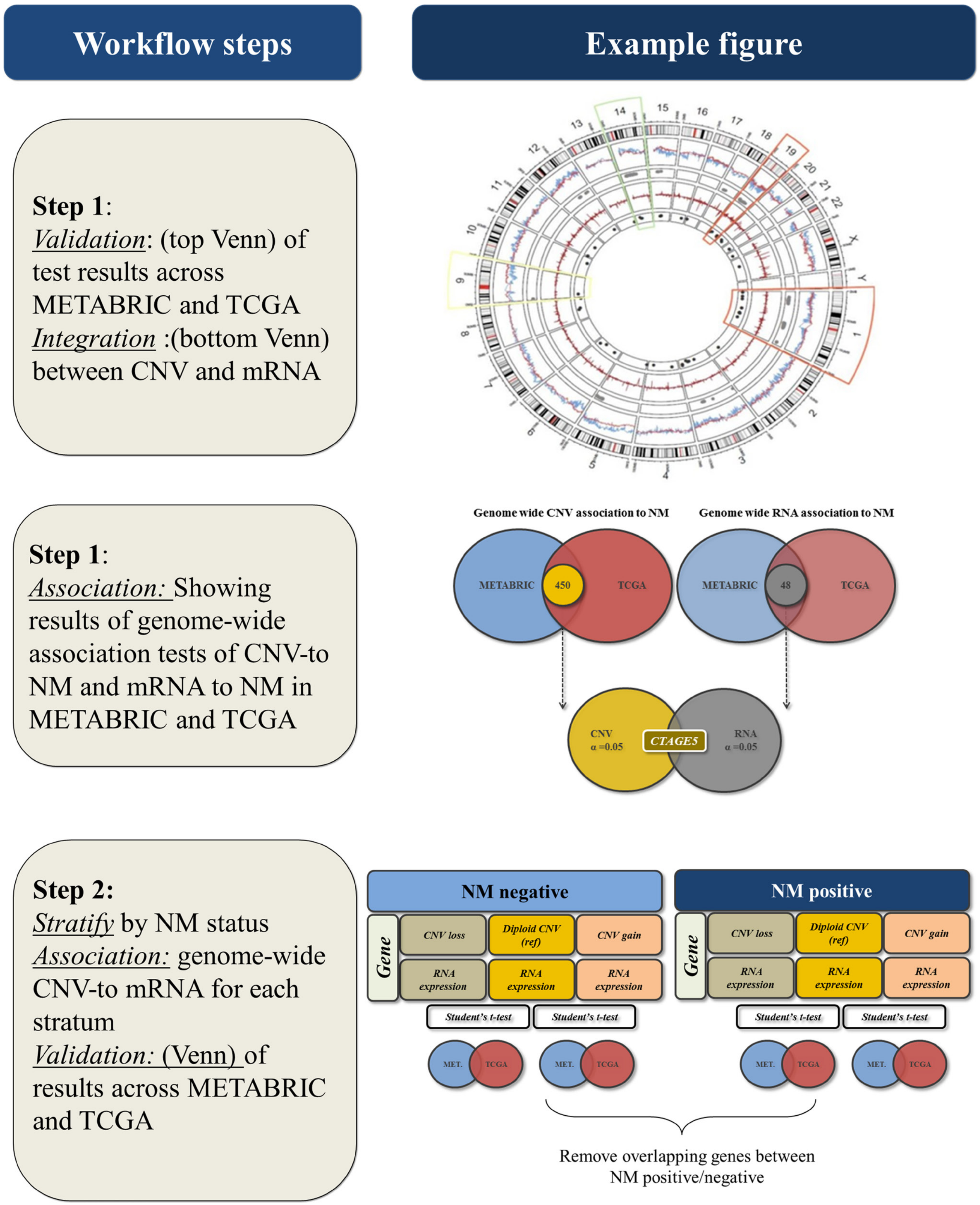

Supplementary Figure 2: Workflow diagram of step 1 and step 2 in analysis approach.

	METABRIC CNV	METABRIC mRNA	TCGA CNV	TCGA mRNA
Model	Logistic regression	Linear regression	Logistic regression	Linear regression
Response	Nodal metastasis yes/no	Nodal metastasis yes/no	Nodal metastasis yes/no	Nodal metastasis yes/no
Predictor	Genome wide CNV (log2 ratio)	Genome wide mRNA (log fold change)	Genome wide CNV (log2 ratio)	Genome wide mRNA (log fold change)
Covariables	Tumor Grade<br>Tumor Size<br>Age at Diagnosis<br>Race	Tumor Grade<br>Tumor Size<br>Age at Diagnosis<br>Race	Molecular Subtype<br>Tumor Size<br>Age at Diagnosis<br>Race	Molecular Subtype<br>Tumor Size<br>Age at Diagnosis<br>Race

Supplementary Figure 3: Description of genome wide association models used in step 1.

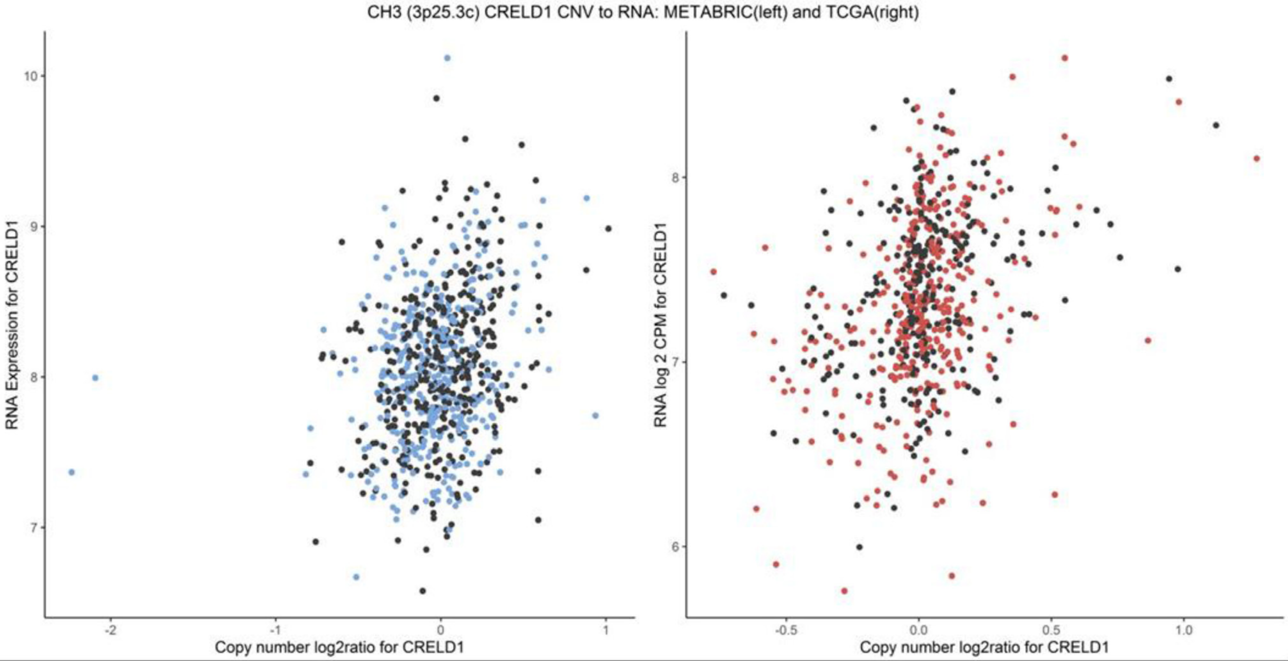

Association test for CNV-driven RNA in CRELD1 (node positives only)

	CNV gains (% patients)	Diploid (% patients)	CNV loss (% patients)	CNV gain mean RNA	Diploid mean RNA	CNV loss mean RNA	FDR-adj. p-value
**METABRIC**	4%	90%	5%	8.56	8.03	7.72	0.04
**TCGA**	4%	91%	5%	7.78	7.31	6.93	0.002

Supplementary Figure 4: CNV-driven changes in CRELD1 RNA for both TCGA and METABRIC, including Student's *t*-test of mean differences in RNA values by CNV status, as well as proportion of patients by CNV status. Black points are NM-negative; red and blue points are NM-positive.

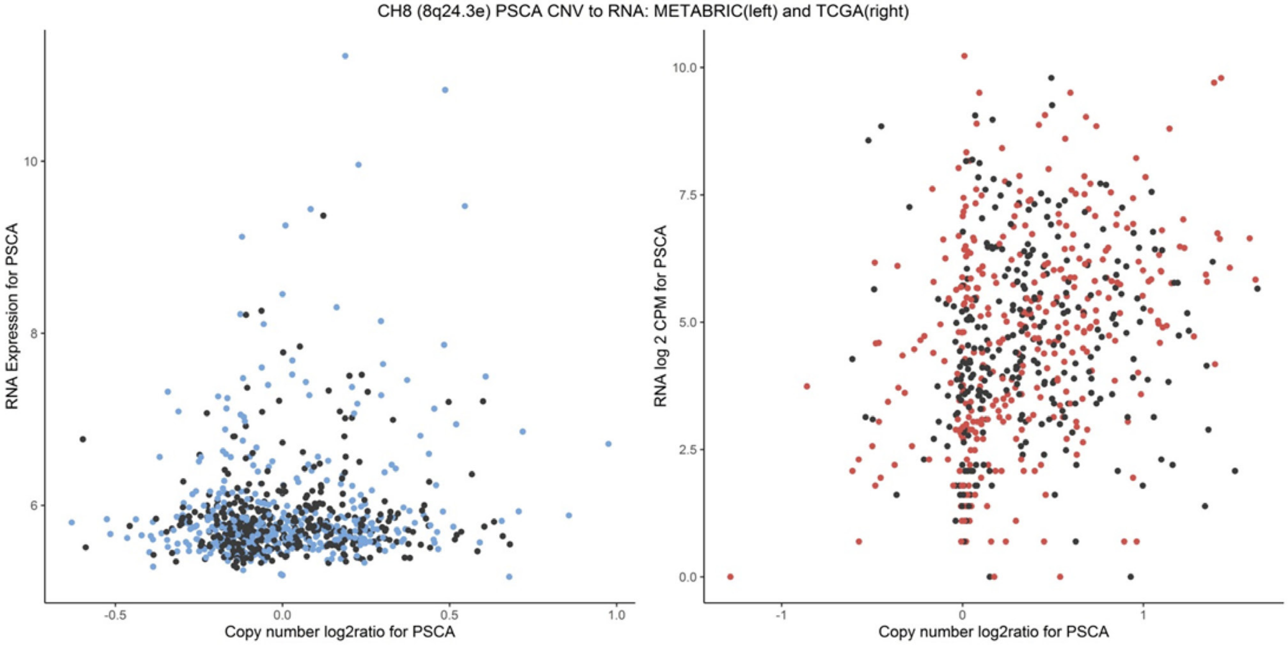

Association test for CNV-driven RNA in PSCA (node positives only)

	CNV gains (% patients)	Diploid (% patients)	CNV loss (% patients)	CNV gain mean RNA	Diploid mean RNA	CNV loss mean RNA	FDR-adj. p-value
**METABRIC**	6%	92%	2%	6.54	5.95	5.74	0.08
**TCGA**	41%	55%	4%	5.45	4.35	2.84	7.13E-08

Supplementary Figure 5: CNV-driven changes in PSCA RNA for both TCGA and METABRIC, including Student's *t*-test of mean differences in RNA values by CNV status, as well as proportion of patients by CNV status. Black points are NM-negative; red and blue points are NM-positive.

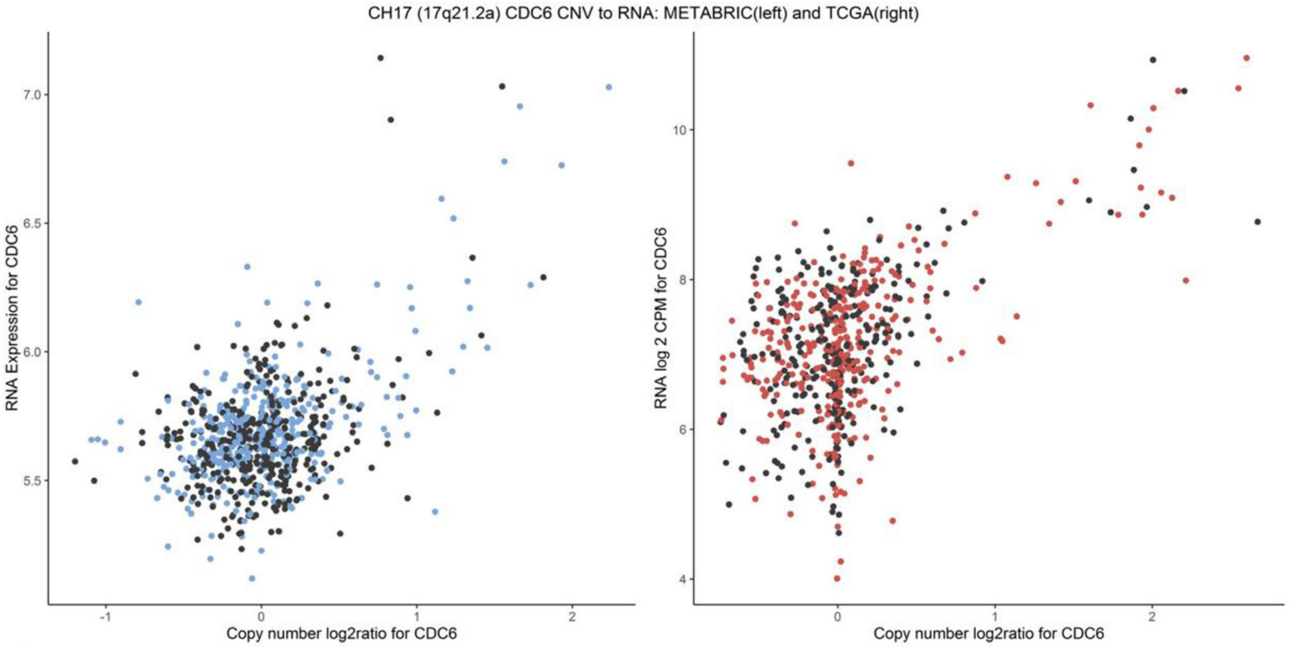

Association test for CNV-driven RNA in CDC6 (node positives only)

	CNV gains (% patients)	Diploid (% patients)	CNV loss (% patients)	CNV gain mean RNA	Diploid mean RNA	CNV loss mean RNA	FDR-adj. p-value
**METABRIC**	11%	80%	9%	5.99	5.66	5.59	4.55E-06
**TCGA**	11%	78%	10%	8.62	6.98	6.80	5.05E-11

Supplementary Figure 6: CNV-driven changes in CDC6 RNA for both TCGA and METABRIC, including Student's *t*-test of mean differences in RNA values by CNV status, as well as proportion of patients by CNV status. Black points are NM-negative; red and blue points are NM-positive.

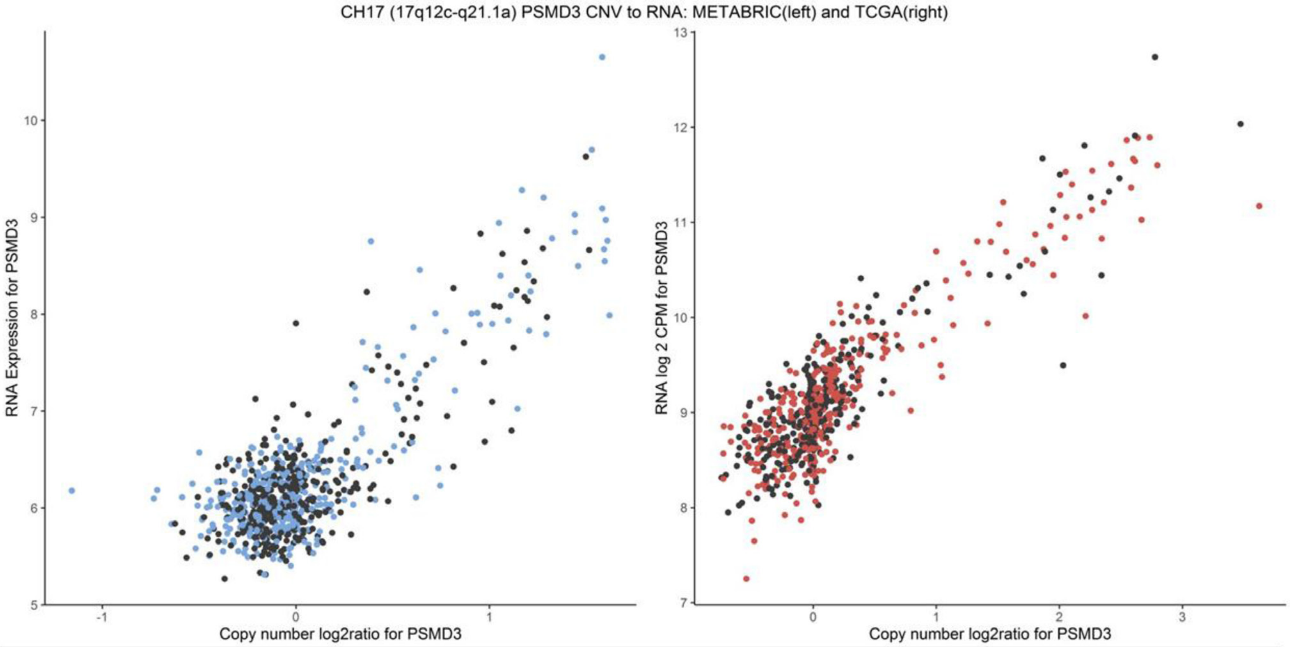

Association test for CNV-driven RNA in PSMD3 (node positives only)

	CNV gains (% patients)	Diploid (% patients)	CNV loss (% patients)	CNV gain mean RNA	Diploid mean RNA	CNV loss mean RNA	FDR-adj. p-value
**METABRIC**	13%	81%	6%	7.90	6.09	5.99	2.48E-15
**TCGA**	17%	74%	9%	10.52	8.98	8.53	9.48E-23

Supplementary Figure 7: CNV-driven changes in PSMD3 RNA for both TCGA and METABRIC, including Student's *t*-test of mean differences in RNA values by CNV status, as well as proportion of patients by CNV status. Black points are NM-negative; red and blue points are NM-positive.

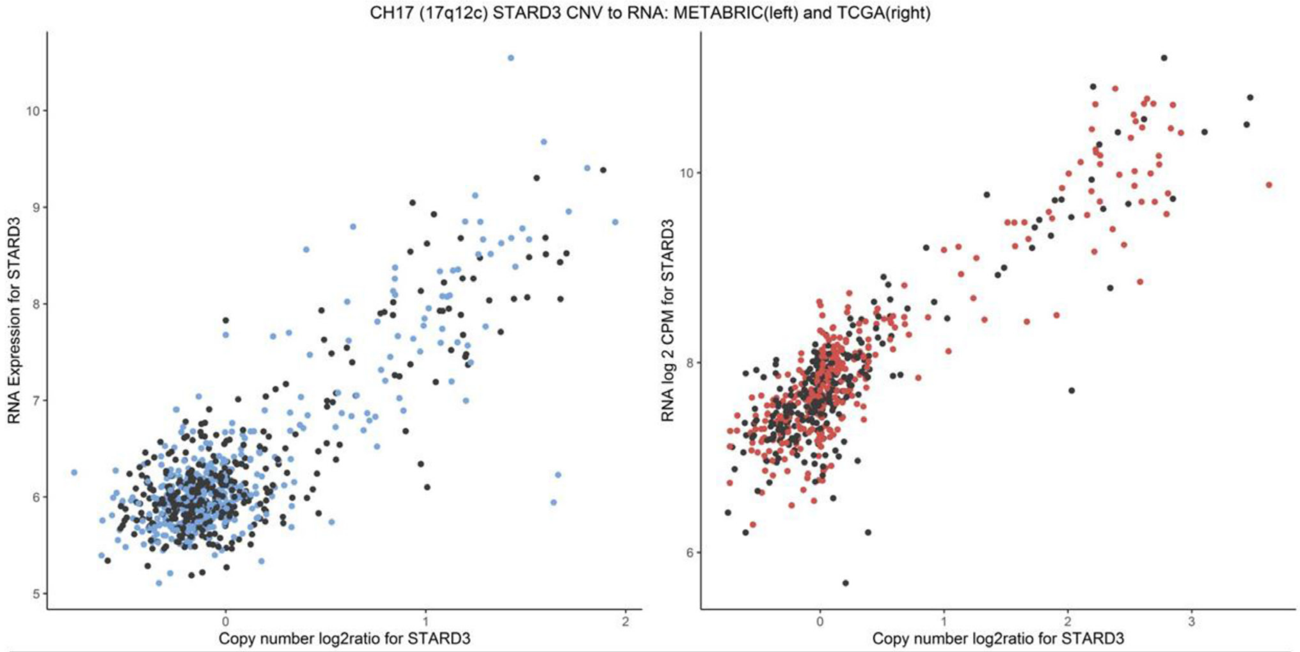

Association test for CNV-driven RNA in STARD3 (node positives only)

	CNV gains (% patients)	Diploid (% patients)	CNV loss (% patients)	CNV gain mean RNA	Diploid mean RNA	CNV loss mean RNA	FDR-adj. p-value
**METABRIC**	17%	77%	6%	7.84	6.03	5.85	1.09E-22
**TCGA**	20%	71%	8%	9.44	7.69	7.20	1.36E-26

Supplementary Figure 8: CNV-driven changes in STARD3 RNA for both TCGA and METABRIC, including Student's *t*-test of mean differences in RNA values by CNV status, as well as proportion of patients by CNV status. Black points are NM-negative; red and blue points are NM-positive.

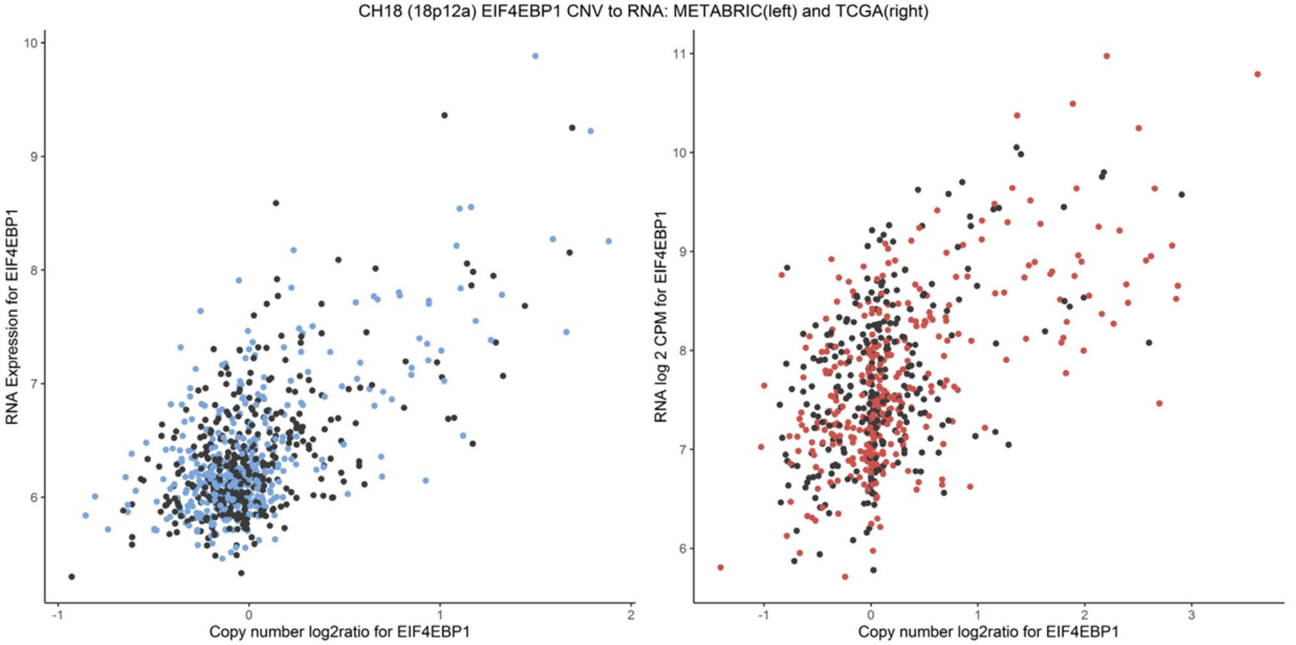

Association test for CNV-driven RNA in EIF4EBP1 (node positives only)

	CNV gains (% patients)	Diploid (% patients)	CNV loss (% patients)	CNV gain mean RNA	Diploid mean RNA	CNV loss mean RNA	FDR-adj. p-value
**METABRIC**	10%	84%	5%	7.44	6.28	6.06	3.20E-10
**TCGA**	24%	63%	11%	8.48	7.51	7.16	8.97E-16

Supplementary Figure 9: CNV-driven changes in EIF4EBP1 RNA for both TCGA and METABRIC, including Student's *t*-test of mean differences in RNA values by CNV status, as well as proportion of patients by CNV status. Black points are NM-negative; red and blue points are NM-positive.

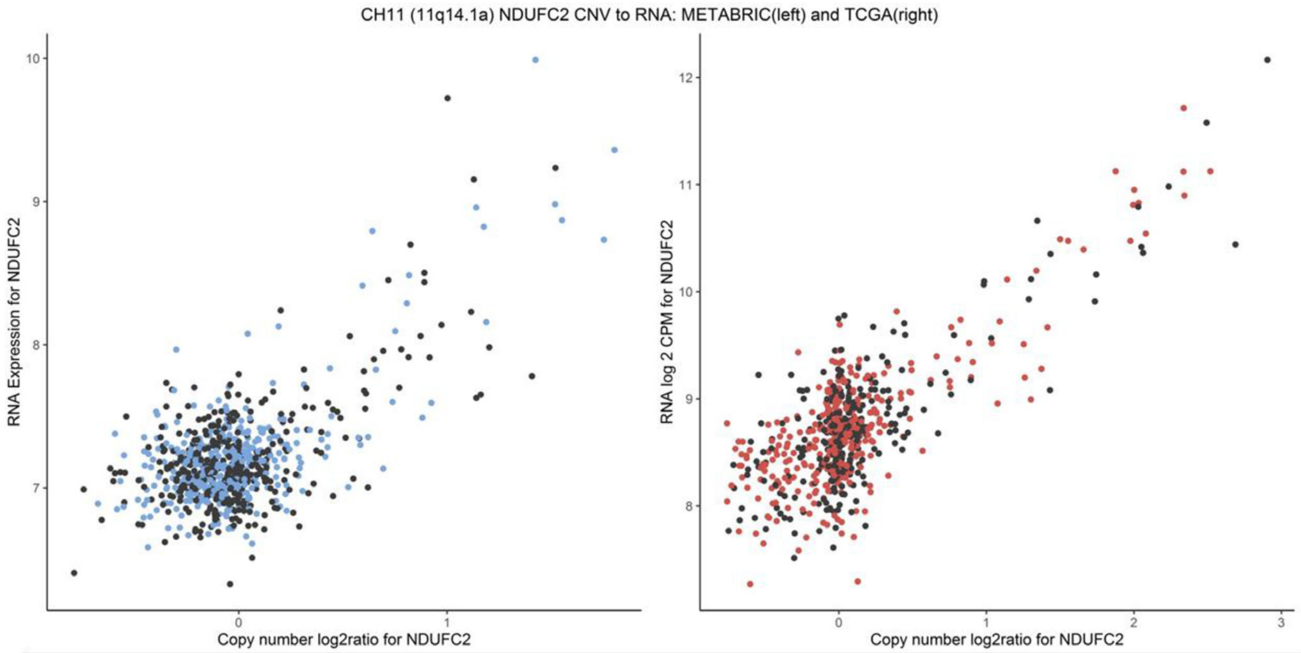

Association test for CNV-driven RNA in NDUFC2 (node positives only)

	CNV gains (% patients)	Diploid (% patients)	CNV loss (% patients)	CNV gain mean RNA	Diploid mean RNA	CNV loss mean RNA	FDR-adj. p-value
**METABRIC**	7%	87%	5%	8.07	7.15	7.03	8.82E-06
**TCGA**	12%	76%	12%	9.78	8.64	8.27	4.43E-11

Supplementary Figure 10: CNV-driven changes in NDUFC RNA for both TCGA and METABRIC, including Student's *t*-test of mean differences in RNA values by CNV status, as well as proportion of patients by CNV status. Black points are NM-negative; red and blue points are NM-positive.

Supplementary Table 1: CNV copy loss. Results of CNV association testing consistent *p*-value and direction in both METABRIC and TCGA - final table of CNV losses associated with NM. See Supplementary_Table_1

Supplementary Table 2: CNV copy gain. Results of CNV association testing consistent *p*-value and direction in both METABRIC and TCGA - final table of CNV gains associated with NM. See Supplementary_Table_2

Supplementary Table 3: RNA low

gene	Start	End	Chromosome	Cytoband	logFC_ METABRIC	pvalMETABRIC	logFC_ TCGA	pvalTCGA
RPL22	6245558	6245607	1	1p36.31b	−0.09	0.0465	−0.13	0.0042
ZSCAN20	33734342	33734391	1	1p35.1a	−0.04	0.0265	−0.12	0.0381
KTI12	52498039	52498088	1	1p32.3e	−0.05	0.0370	−0.11	0.0029
VPS45	148305965	148388793	1	1q21.2a	−0.06	0.0441	−0.09	0.0375
MRPL9	150002664	150005754	1	1q21.3a	−0.06	0.0300	−0.12	0.0070
ZNF648	180297470	180627573	1	1q25.3c	−0.05	0.0171	−0.34	0.0342
CRYBG3	97660022	97660071	3	3q11.2c	−0.03	0.0090	−0.26	0.0052
ACPP	133518901	133619242	3	3q22.1c	−0.04	0.0376	−0.26	0.0318
SPSB4	142253432	142433371	3	3q23b	−0.03	0.0206	−0.40	0.0046
CP	150422522	150534109	3	3q24f-q25.1a	−0.14	0.0240	−0.95	0.0000
LRAT	155884612	155921949	4	4q32.1a	−0.03	0.0234	−0.39	0.0183
PDCD6	314686	314735	5	5p15.33e	−0.08	0.0120	−0.09	0.0367
RPL10A	35436179	35436211	6	6p21.31c	−0.04	0.0377	−0.13	0.0039
HS3ST5	114490734	116488614	6	6q22.1a	−0.03	0.0407	−0.93	0.0006
BAI1	143542378	143692835	8	8q24.3e	−0.03	0.0178	−0.57	0.0002
CYP2C8	96814630	96814679	10	10q23.33c	−0.07	0.0379	−0.78	0.0001
RELT	72765058	72765107	11	11q13.4b	−0.03	0.0490	−0.17	0.0074
MYO16	108046500	109236915	13	13q33.3c	−0.04	0.0187	−0.54	0.0011
FBXL19	30923055	30948783	16	16p11.2	−0.04	0.0075	−0.11	0.0449
ZNF77	2884594	2884643	19	19p13.3f	−0.05	0.0435	−0.14	0.0051
ZNF614	57208731	57208780	19	19q13.33e	−0.05	0.0149	−0.16	0.0066
AHCY	32332113	32332162	20	20q11.22a	−0.10	0.0397	−0.19	0.0004
SYN3	31238824	31238873	22	22q12.3a	−0.03	0.0020	−0.26	0.0402

Results of RNA association testing consistent *p*-value and direction in both METABRIC and TCGA - final table of RNA losses associated with NM.

Supplementary Table 4: RNA high

gene	Start	End	Chromosome	Cytoband	logFC_METABRIC	pvalMETABRIC	logFC_TCGA	pvalTCGA
MAGI3	114000000	114000000	1	1p13.2c-p13.2b	0.03	0.0167	0.18	0.0084
NRAS	115000000	115000000	1	1p13.2a	0.07	0.0426	0.14	0.0292
PEAR1	155000000	155000000	1	1q23.1a	0.04	0.0410	0.15	0.0467
DLX1	173000000	173000000	2	2q31.1d	0.14	0.0107	0.59	0.0097
ZMAT3	180000000	180000000	3	3q26.32c	0.10	0.0363	0.15	0.0285
TMEM156	38968445	38968494	4	4p14c	0.11	0.0214	0.39	0.0091
SAMD5	148000000	148000000	6	6q24.3b	0.05	0.0395	0.36	0.0030
HEY1	80838954	80839003	8	8q21.13a	0.12	0.0298	0.26	0.0032
PTPLAD2	20993986	20994035	9	9p21.3d	0.09	0.0485	0.31	0.0005
SYT8	1814854	1814896	11	11p15.5b	0.05	0.0457	0.40	0.0486
TNNI2	1819387	1819436	11	11p15.5b	0.09	0.0106	0.29	0.0324
KCTD10	110000000	110000000	12	12q24.11b	0.08	0.0372	0.10	0.0032
NOVA1	26949191	26949240	14	14q12b	0.16	0.0156	0.52	0.0018
FAM177A1	34585403	34585420	14	14q13.2a	0.06	0.0453	0.15	0.0019
CTAGE5	38805341	38805390	14	14q21.1b	0.06	0.0120	0.10	0.0386
MAPKBP1	39907042	39907091	15	15q15.1c	0.03	0.0432	0.08	0.0359
PLDN	43688090	43688139	15	15q21.1a	0.08	0.0423	0.10	0.0051
SHC4	46903514	46903563	15	15q21.1d	0.12	0.0465	0.38	0.0336
ZFP90	67158424	67158473	16	16q22.1c	0.07	0.0139	0.08	0.0381
GPR172B	4876842	4876891	17	17p13.2b	0.07	0.0237	0.37	0.0099
MAST1	12846639	12846684	19	19p13.13c	0.06	0.0067	0.35	0.0239
SLC17A7	54624645	54624694	19	19q13.33b	0.03	0.0058	0.29	0.0067
NAPB	23303396	23303445	20	20p11.21c	0.10	0.0214	0.22	0.0042
SYNJ1	32925209	32925258	21	21q22.11b	0.05	0.0384	0.13	0.0073
TRO	54957643	54957692	X	Xp11.21a	0.10	0.0483	0.29	0.0026

Results of RNA association testing consistent *p*-value and direction in both METABRIC and TCGA - final table of RNA gains associated with NM.

Supplementary Table 5: CNV driven RNA loss. Results of Student's *t*-test comparing variation in RNA expression in CNV copy loss versus diploid/neutral in both groups of NM. See Supplementary_Table_5

Supplementary Table 6: CNV driven RNA gain. Results of Student's *t*-test comparing variation in RNA expression in CNV copy gain versus diploid/neutral in both groups of NM. See Supplementary_Table_6

Supplementary Table 7: CNV driven RNA change. Results of Student's *t*-test comparing variation in RNA expression in CNV copy gain as well as copy loss versus diploid/neutral in both groups of NM. See Supplementary_Table_7

Supplementary Table 8: CNV driven changes in protein and methylation

Gene	NM positive (n = 357)			
	CNV to protein		CNV to methylation	
	effect	p-value	effect	p-value
CRELD1	0.30	3.32E-06	−0.15	0.02
EIF4EBP1	0.61	<2.2e-16	−0.15	0.02
PSMD3	0.89	<2.2e-16	0.36	9.77E-09
STARD3	0.91	<2.2e-16	−0.10	0.1245

Gene	NM negative (n = 293)			
	CNV to protein		CNV to methylation	
	effect	p-value	effect	p-value
CRELD1	0.47	1.59E-10	−0.07	0.36
EIF4EBP1	0.56	4.55E-15	−0.12	0.13
PSMD3	0.90	<2.2e-16	0.40	9.08E-08
STARD3	0.89	<2.2e-16	−0.13	0.09

Results of Pearson correlation between CNV changes and protein as well as CNV changes and methylation for the genes CRELD1, EIF4EBP1, PSMD3, and STARD3 in separate groups NM-positive women and NM-negative women.
